# DNA flowerstructure co-localizes with human pathogens in infected macrophages

**DOI:** 10.1093/nar/gkaa341

**Published:** 2020-05-13

**Authors:** Oskar Franch, Camino Gutiérrez-Corbo, Bárbara Domínguez-Asenjo, Thomas Boesen, Pia Bomholt Jensen, Lene N Nejsum, Josephine Geertsen Keller, Simon Pagaard Nielsen, Prakruti R Singh, Rajiv Kumar Jha, Valakunja Nagaraja, Rafael Balaña-Fouce, Yi-Ping Ho, Rosa María Reguera, Birgitta Ruth Knudsen

**Affiliations:** 1 Department of Molecular Biology and Genetics, Aarhus University, Aarhus, Denmark; 2 Interdisciplinary Nanoscience Center (iNANO), Aarhus University, Aarhus, Denmark; 3 Department of Biomedical Science, University of León, León, Spain; 4 DANDRITE, Nordic EMBL Partnership for Molecular Medicine, Department of Molecular Biology and Genetics, Aarhus University, Aarhus, Denmark; 5 Department of Clinical Medicine, Aarhus University, Aarhus, Denmark; 6 Department of Microbiology and Cell Biology, Indian Institute of Science & Jawaharlal Nehru Centre for Advanced Scientific Research, Bangalore, India; 7 Department of Biomedical Engineering, The Chinese University of Hong Kong, Hong Kong SAR; 8 Centre for Novel Biomaterials, The Chinese University of Hong Kong, Hong Kong SAR

## Abstract

Herein, we characterize the cellular uptake of a DNA structure generated by rolling circle DNA amplification. The structure, termed nanoflower, was fluorescently labeled by incorporation of ATTO488-dUTP allowing the intracellular localization to be followed. The nanoflower had a hydrodynamic diameter of approximately 300 nanometer and was non-toxic for all mammalian cell lines tested. It was internalized specifically by mammalian macrophages by phagocytosis within a few hours resulting in specific compartmentalization in phagolysosomes. Maximum uptake was observed after eight hours and the nanoflower remained stable in the phagolysosomes with a half-life of 12 h. Interestingly, the nanoflower co-localized with both *Mycobacterium tuberculosis* and *Leishmania infantum* within infected macrophages although these pathogens escape lysosomal degradation by affecting the phagocytotic pathway in very different manners. These results suggest an intriguing and overlooked potential application of DNA structures in targeted treatment of infectious diseases such as tuberculosis and leishmaniasis that are caused by pathogens that escape the human immune system by modifying macrophage biology.

## INTRODUCTION

DNA structures present several advantages for drug delivery purposes. These include biocompatibility, safety, stability and the ease by which entities with different shape, size and functionalizations can be designed (1). Cellular delivery of unfunctionalized or various functionalized DNA structures to cell lines or even in whole organisms have been demonstrated in a large number of studies (2–13). Interestingly, in the context of drug delivery purposes, the rigid or compact nature of DNA structures appears to make them more prone for entering living cells than linear single- or double-stranded DNA (14). For example, efficient cellular uptake of Spherical Nucleic Acids (SNAs) was demonstrated to be facilitated by scavenger receptors (15,16). Consistently, we observed uptake of a pristine octahedral DNA nanocage in COS-1 cells transfected with a scavenger receptor (Lox1) expressing plasmid but not in un-transfected COS-1 (17) or HEK293T cells (18). The Fan and Tuberfield groups, on the other hand, demonstrated uptake of a pristine DNA tetrahedron in un-transfected HeLa and HEK293T cells (19,20) and studies suggest that the ‘pointy-ends’ in tetrahedral structures may facilitate internalization (21,22).

Once internalized, the cellular stability of DNA structures is rather high varying between 24 and 48 h depending on the identity of the structure (20,23,24). Unless targeted to other organelles, most DNA structures are directed to the phagolysosomes of treated cells (17,19,23,25,26). This localization presents an obvious advantage, when it comes to treatment of infectious diseases caused by intracellular pathogens that use phagolysosomes or immature phagosomes in macrophages as a safe haven to escape the host immune system and reproduce. Examples of such pathogens are the bacteria *Mycobacteria tuberculosis* and *Salmonella* sp. or eukaryotic parasites such as *Trypanosoma cruzi* and *Leishmania* sp. that all cause severe, in some cases deathly, human diseases (27–30). Common for these pathogens is, that they are engulfed by macrophage phagocytosis. Normally, phagosomes fuse with lysosomes and mature into phagolysosomes with an extreme acidic environment that degrade the invading pathogen (31,32). However, pathogens like the above-mentioned species survive by delaying or circumventing the maturation of phagosomes (32). Roughly speaking, pathogens that can survive phagocytosis can be subdivided into three main groups. Members of the first group (group 1) prevent phagosome maturation and includes *M. tuberculosis* and *Salmonella* sp. (31,32). Members of the second group (group 2), including *Leishmania spp*. delay phagosome maturation long enough to adapt to the extreme environment (32). Finally, members of the third group (group 3) such as *Trypanosoma cruzi*, only localize to the phagosomes for a limited amount of time, before they escape to the cytosol (32).

The biology of intracellular pathogens complicates the treatment of the related diseases such as tuberculosis and leishmaniasis dramatically. The increased frequencies of multi-drug-resistant subtypes of these diseases are therefore a subject of outmost concern worldwide (33) (https://www.who.int/emergencies/ten-threats-to-global-health-in-2019),^34^ (https://www.who.int/features/qa/79/en/). Attempts to improve treatment options have included the investigation of various drug carriers. However, recent studies of targeted treatment utilizing a variety of nanoparticles including polymeric micelle, carbon nanotubes, lipid and polymeric nanoparticles have only demonstrated moderate success primarily due to toxicity inherent to the utilized nanoparticles combined with low drug incorporation (35,36).

The high biocompatibility of DNA structures combined with their potential accumulation in the phagolysosomes suggests that they may be successful as carriers for targeted treatment of infectious diseases caused by either the group 1 or 2 pathogens mentioned above. However, the modulation of phagosome maturation inflicted by pathogens may influence the fate of the DNA structures in infected cells. Hence, phagolysosomal localization in uninfected cells does not necessarily imply co-localization between DNA structures and pathogens in infected macrophages. Therefore, in the present study we address the cellular uptake and potential co-localization of DNA structures with macrophage residing pathogens. For this purpose, *M. tuberculosis* and *Leishmania infantum* were selected as model pathogens from the groups 1 and 2, respectively. A DNA nanoflower (NF) structure originally described by the Tan group was chosen as the DNA structure for our studies (10). NF structures were previously characterized as spherical entities with diameters from 200 nm to 4 μm when analyzed by scanning electron microscopy (SEM) (10). NF self assembles from long tandem repeats of DNA generated by Rolling Circle Amplification (RCA) of circular 63- to 123-mer oligonucleotide templates (37). The particular NF used in the current study was generated from a 97-mer template. Although NFs are characterized by a higher degree of heterogeneity than tile- or origami-based DNA structures, NFs can be produced with relatively well-defined sizes and structures (11,38,39). Of particular interest for real-life applications, NF present considerable advantages associated to the ease by which they can be functionalized, labeled and produced in high quantities from a single template and primer oligonucleotide in a simple one-step procedure at reasonably low costs. In the current study we successfully demonstrate specific uptake of a fluorescently labeled NF in macrophages, accumulation of the NF in phagolysosomes and co-localization with both *L. infantum* or *M. tuberculosis* in infected cells. This illustrates the potential of employing such DNA structure as a carrier for specific targeted treatment of diseases such as leishmaniasis and tuberculosis.

## MATERIALS AND METHODS

### Enzymes and reagents

T4 polynucleotide kinase (PNK), T4 DNA ligase, and bovine serum albumin (BSA) (New England Biolabs), ATP (Amersham Biopharma) and Phi29 DNA Polymerase (Thermofisher) were purchased from the vendors and used according to the manufactures’ protocols. Synthetic DNA substrates were purchased from LGC Biosearch Technologies. Cell lines were purchased from ATCC or acquired from collaborators listed in the acknowledgement. Culture media, solutions and reagents for cells were purchased from Gibco.

### Production, labeling and characterization of NF

The NF have been produced using the template oligonucleotide (OL1). OL1 was 5′-phosphorylated by PNK for a final concentration of 0.1 μM and 0.1 units/μl, respectively. The 5′-phosphorylated OL1 was performed for 1 h using 1× PNK buffer supplied by the manufacturer and 500 μM ATP. Following the 5′-phosphorylation, a primer oligonucleotide (OL2) was added for a final concentration of 0.5 μM, before all secondary structure in the DNA was disrupted by incubated at 95°C for 10 min. The solution was allowed to cool to room temperature, before fresh ATP and T4 DNA Ligase were added to the solution obtaining a concentration of 100 μM and 0.4 units/μl and reaction proceeded for 16 hours at room temperature. The resulting circular template with primer was either used directly for RCA or stored at –20°C. RCA was performed using final concentration of 0.1 units/μl Phi29 DNA polymerase and 80 μM of nucleotides. After incubating 16 h at 30°C, RCA was stopped by heating the reaction to 80°C for 10 min. For incorporation of ATTO488, aminoallyl-dUTP-XX-ATTO-488 (Jena Bioscience) was included in the RCA reaction mixture in a 1:20 molar relationship with dTTP. ATTO488-NF and NF were ethanol precipitated by adding 1/10 volume of 4 M NaCl and three times volume of 96% ethanol, before incubating the samples at –20°C for 12 h. ATTO488-NF and NF were stored at –20°C until usage. Ethanol precipitation was performed to obtain sterile conditions for uptake experiments and to remove background from unincorporated fluorescently labeled dUTP. The resulting pellet was resuspended in water for analyses using SEM and Transmission Electron Microscopy (TEM) and in sterile PBS for other experiments. Quality control and concentration determination were addressed both by measuring DNA absorbance using GeneQuant 1300 spectrophotometer and by analyzing the product in a 1% (w/v) agarose gel. Pictures of fluorescently labeled DNA bands in agarose gels were obtained using a Typhoon Scanner FA 9500. DNA bands were subsequently visualized by ethidium bromide (EtBr) staining and Image Lab software from Bio-Rad was used for quantification.

**Table:** 

OL1	TAGGGTTAGGGAAAACTGTGAAGATCGCTTATAAACCCTAACCCTAACCCTAACCCTAAACCTCAATGCTGCTGCTGTACTACAAAAGGGTTAGGGT
OL2	ATAAGCGATCTTCACAGT

### Cell lines

C2C12, RAW264.7 and THP-1 cells were cultured in Dulbecco's modified Eagle's medium—high glucose, supplemented with 10% (v/v) heat inactivated fetal bovine serum (FBS) and 1% (v/v) antibiotic cocktail (100 U/ml penicillin, and 100 mg/ml streptomycin) in a HERA cell incubator at 37°C with 5.6% CO_2_. Cells were kept below passage 20. THP-1 monocytes at 6 × 10^5^ cells/ml were differentiated into macrophages using 200 nM of phorbol 12-myristate 13-acetate (PMA) for 48 h at 37°C and 5% CO_2_. THP-1 cells were subsequently allowed to fully differentiate in medium without PMA for 4 days, before subjected to experiments.

### Mice and parasites

The animal research described in this manuscript complies with Spanish Act (RD 53/2013) and European Union Legislation (2010/63/UE). The used protocols were approved by the Animal Care Committee of the University of León (Spain), project license number (JMJ/bb). Female BALB/c mice (6–8 weeks old) were obtained from Janviers Lab (France) and housed in specific-pathogen-free facilities for this study. The infrared fluorescence-emitting strain iRFP+ *L. infantum* was previously described (40). iRFP+ *L. infantum* promastigotes were routinely cultured at 26°C in M199 medium supplemented with 25 × 10^−3^ M HEPES pH 6.9, 10 × 10^−3^ M glutamine, 7.6 × 10^−3^ M hemin, 0.1 × 10^−3^ M adenosine, 0.01 × 10^−3^ M folic acid, RPMI 1640 vitamin mix (Sigma), 10% (v/v) heat inactivated FBS, and antibiotic cocktail (50 U/m; penicillin, 50 mg/ml streptomycin).

### Infection of macrophage cultures

Freshly WT and iRFP+ *L. infantum* amastigotes isolated from infected BALB/c mice spleens were added to macrophage cultures at an infection ratio of 5:1 and incubated at 37°C and 5% CO_2_ in complete RPMI medium for 6 hours. To remove non-internalized parasites, cultures were washed 5 times with 37°C pre-warmed PBS and cultured in complete RPMI medium at 37°C in a 5% CO_2_ atmosphere. Macrophages were incubated with samples as described in main text. Quantification of the co-localization between iRFP+ *L. infantum* and ATTO488-NF was performed by counting co-localization event in representative pictures from the confocal microscopy analysis.

THP-1 macrophages were infected with *M. tuberculosis* (H37Rv strain) expressing Td tomato fluorescent protein, at 1:10 multiplicity of infection (MOI) 6 hours post infection, THP-1 macrophages were incubated with gentamycin (50 μg/ml) for 1 h. Macrophages were then washed with PBS and complete RPMI medium was added simultaneously with 8.4 ng/ml of ATTO488-NF. After 8 h incubation at 37°C in CO_2_ incubator, cells were fixed with 4% paraformaldehyde for 15 min. Nuclei were stained with DAPI and observed under Leica TCS SP8 microscope at 63× magnification.

### Cellular uptake studies

For flow cytometry 12 × 10^4^ and 8 × 10^4^ of RAW264.7 and C2C12 cells, respectively, were seeded in standard 6-well plates (Sarstedt) in a volume of 1 ml. After 24 h, samples were added to the cells. Cells were incubated for 8 h at 37°C, before they were washed twice with 37°C pre-warmed PBS and analyzed using Beckman Coulter's CytoFLEX LX Flow Cytometer. Subsequently, data from single cells were isolated using FlowJo software and fluorescence from NF-ATTO488 in single cells were measured using the FITC channel. The threshold for positive uptake was defined by the 1% cells of the negative control (incubated without NF-ATTO488) that gave most signal in the FITC channel. This threshold is indicated as a vertical line in Figure 2. The percentage of positive cells defined by an ATTO488 emission above this threshold was used to estimate the uptake efficiency in the specific cell lines. Statistical significance between populations distributions was tested using a chi-squared *T*(χ) test with FlowJo software. A value *T*(χ) > 4 was accepted as significantly different. A value *T*(χ) > 4 implies that the two distributions are the same with <1% probability (*P* < 0.01) (i.e. 99% confidence that the distributions are different).

For confocal analysis 200 μl of RAW264.7, THP-1 monocytes and C2C12 cells were seeded at a density of 6 × 10^4^, 20 × 10^4^ and 5 × 10^4^ cells/ml, respectively, in eight-well ibidi coated plates. THP-1 monocytes were differentiated as described above. After incubation with samples for 8 h at 37°C, cells were washed twice with 37°C pre-warmed PBS. Cells were stained with 1 × 10^−6^ M Cresyl Violet for 5 min and 2.5 μg/ml Hoechst 33342 dissolved in culture medium. Images were obtained using ZEISS ZEN Imaging Software System. The Scatterplot of co-localization was made using Coloc2 software (version 3.0.5) in ImageJ 1.52p.

### Scanning electron microscopy and transmission electron microscopy

NF were prepared as described above. Prior to TEM analyses, formaldehyde crosslinking was performed by incubating 2.5 μg/ml of NF in a solution of 3% formaldehyde and 1× PBS buffer at room temperature for 1 h, before the crosslinking reaction was terminated by adding ethanolamine for a concentration of 13 mM. Finally, the NF was ethanol precipitated, as described above, and resuspended in water for a concentration of 2.5 μg/ml.

For SEM, a few microliters of the NF sample was applied to a piece of silicon wafer and left for drying. SEM was done on a Versa 3D (Thermo Fisher Scientific) operated at 5 kV 13pA and using a stage tilted to 45°.

For TEM, 3 μl of sample was applied to a 400-mesh collodion and carbon coated copper grid that had been glow-discharged and the grids were subsequently blotted and stained three times with 2% uranyl formate. Micrographs were collected on a Tecnai G2 Spirit TWIN electron microscope (Thermo Fisher Scientific) operated at 120 kV and using a Tietz TemCam-F416 CMOS camera at a nominal magnification of 67 000× with a pixel size of 1.57 Å.

## RESULTS

### Production and characterization of the DNA nanoflower structure

**Figure 1.**
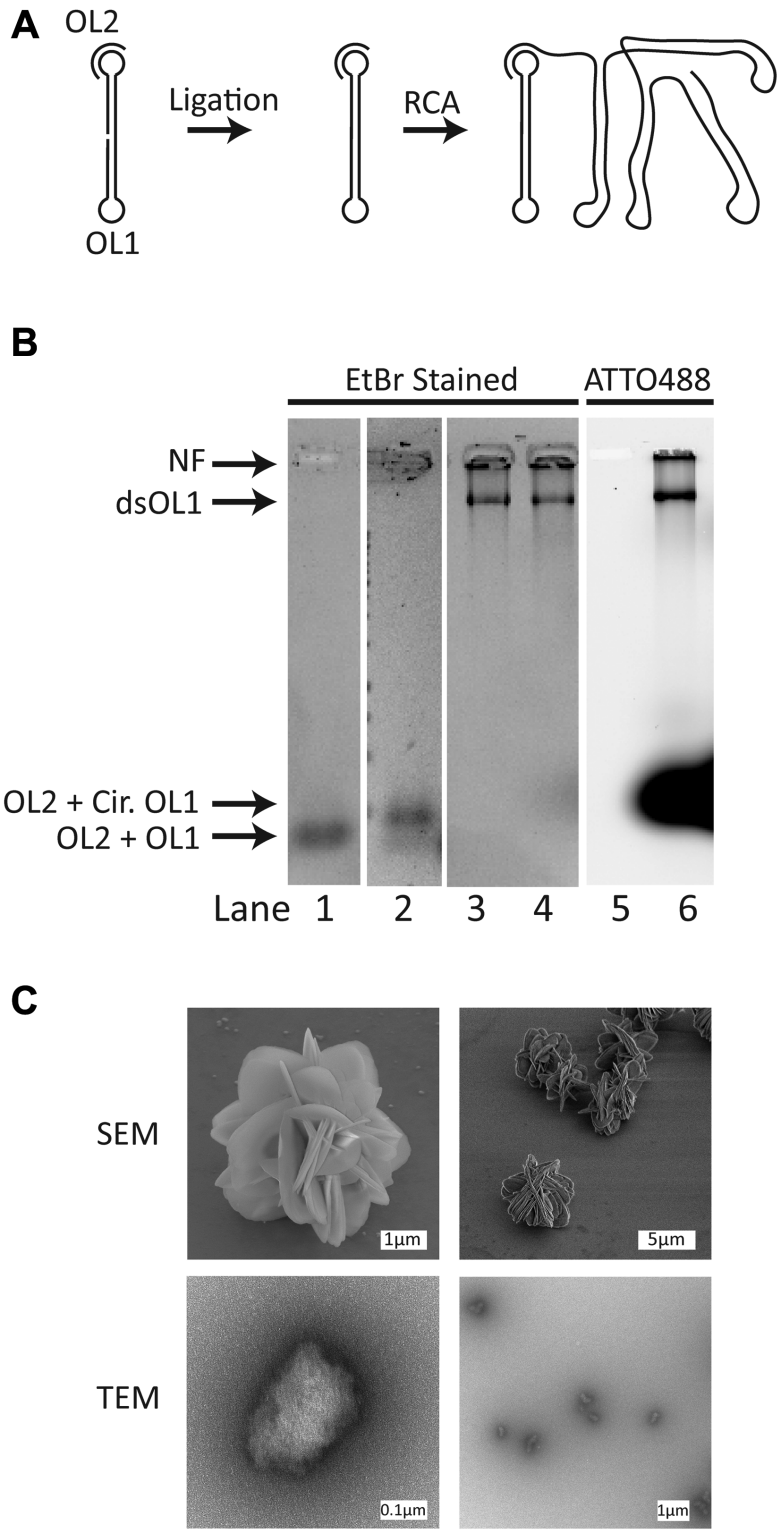
(**A**) Shows the secondary structure of the DNA template hybridized with a DNA primer. The dumbbell structure of the DNA template is initially ligated into a covalently closed circle of single stranded DNA (ssDNA). Subsequently, the DNA circle is subjected to RCA by elongating the hybridized primer. The RCA yields ssDNA consisting of long tandem repeat sequences. (**B**) Gel electrophoretic analysis of the production of the NF with and without fluorescent labeling with ATTO488-dUTP. Lane 1 shows the mobility of OL2 hybridized to OL1 prior to ligation. Lane 2 shows the mobility of OL2 hybridized to OL1 after ligation. Lanes 3 and 4, the NF without or with incorporated dUTP labeled ATTO488, respectively. DNA in lanes 1–4 has been visualized using EtBr staining. Lanes 5 and 6 are the same as 3 and 4, but have been visualized using fluorescence from ATTO488 instead. The black arrows indicate the mobility of the NF, dsOL1, circularized OL1 hybridized to OL2 and non-circularized OL1 hybridized with OL2 in the gel. (**C**) SEM and TEM pictures of the NF as indicated to the left of the pictures.

The clear advantage of NFs over tile- or origami-based structures is the ease by which they can be produced in relatively large quantities. In the current study, we used a 97-mer oligonucleotide (termed OL1) with a sequence that folds into a dumbbell-shaped structure (see Figure 1A) as a template for the RCA that generated the NF. This template design was chosen, because it facilitated easy and 100% efficient circularization by ligation (data not shown) rendering purification of the ligated template unnecessary. OL1 was hybridized with an 18-mer oligonucleotide (OL2), which functioned as a primer for the RCA (see Figure 1A), before the ends of OL1 were ligated to generate a closed DNA circle. The NF was produced in an unlabeled and in a labeled form. The labeling was performed by incorporation of ATTO488-dUTP in the RCA products, henceforth the labeled form is termed ATTO488-NF. The reaction products were analyzed by gel-electrophoresis and the products visualized by staining of the gel with EtBr or scanning of ATTO488 fluorescence as indicated on top of the gel pictures (Figure 1B). Ligation resulted in a slight retardation of OL1 compared to the un-ligated oligonucleotide (compare lanes 1 and 2) indicating the generation of a closed DNA circle (marked cir. OL1 in Figure 1B). RCA in the presence of unlabeled or labeled nucleotides resulted in a low-mobility product that was retarded in the slot (lanes 3, 4 and 6 marked NF). This mobility is consistent with the expected mobility of the NF product as reported by others (41). Moreover, a side product marked double stranded DNA (dsDNA) with a slightly faster mobility than the NF product is also evident in lanes 3, 4 and 6. This product most probably represents dsDNA generated by template switching during RCA as described by the Ducani *et al.* from the Högberg laboratory (41).

The dimensions and structure of the produced NF (corresponding to the product retained in the slot in Figure 1B) were analyzed by SEM or TEM. As evident on the top panel of Figure 1C, the NF appeared as a spherical structure with pedal-like components and a diameter of ∼4–5 μm when analyzed in SEM. It is, however, likely that the SEM structure represents a crystalized form of the NF generated during dehydration of the sample. TEM analysis, presented in the bottom panel of Figure 1C, revealed the NF as rounded particles with diameters of ∼200–500 nm. Note that formaldehyde was used to crosslink NF and preserve the structures for electron microscopy analysis and the uranyl formate stain used in TEM is also known to act as a fixative which could further preserve the size and morphology of the NFs upon drying the samples. Similar sizes were observed when analyzing non-crosslinked NF using SEM and by visualization of ATTO488-NF in a fluorescent microscope (data not shown). The size of our NF structure, as revealed by TEM and fluorescent microscopy, is in agreement with similar studies of pristine NFs in the Tan group (10,11). We note that Wei *et al.* reported the generation of a NF with a diameter of 1000–1500 nm using a template of around the same size (98 nucleotides) as ours (97 nucleotides) and by performing the RCA reaction for approximately the same amount of time (25). We attribute the discrepancy between our sizes and the size reported by Wei *et al.* to difference in staining and drying protocol prior to the imaging of their NF, which could lead to increased sizes due to aggregation. We further performed Dynamic Light Scattering analysis of our NF in solution, which confirm that our particles indeed have a hydrodynamic diameter of 324 nm (Supplementary Figure S1), which is within the same size range as the particles observed in the TEM analysis. Carriers with dimensions within the range of 7.2–500 nm are well suited as vessels for drug delivery since they are small enough for fast circulation (42) and diffusion and still large enough to avoid renal exclusion (43).

### Uptake of the NF in mammalian cell lines derived from different tissues

**Figure 2.**
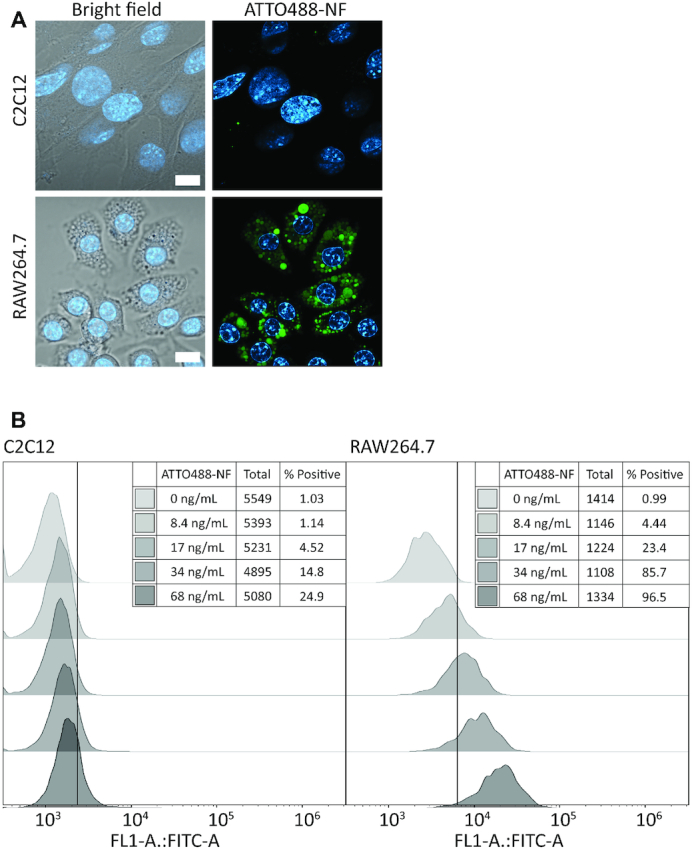
(**A**) Confocal microscopic pictures that illustrate the uptake in the myoblast cell line (C2C12) and in the macrophage cell line (RAW264.7) after incubating the cells with 8.4 ng/ml of ATTO488-NF. Left column are bright field pictures with Hoechst stained nucleus (blue) superimposed. The right column shows the ATTO488-NF (green) and the Hoechst stained nucleus. The white scale bars in the bright field microscopy pictures are 10 μm. (**B**) Flow cytometry analysis of ATTO488-NF uptake in C2C12 and RAW264.7. The histogram shows a representative result out of three individual experiments. The x-axis denotes the fluorescence intensity measured and the y-axis indicates the number of cells. The vertical line through the histograms shows the threshold of the cells positive for taking up ATTO488-NF as defined in the materials and methods section. From top to bottom, the histograms represent cells incubated with 0, 8.4, 17, 34 and 68 ng/ml of ATTO488-NF, respectively. The tables in the top right corner of the histogram-collections, show the total number of cells represented in the histograms and the percent of positive cells. Using FlowJo software, the ATTO488 signal in the cell populations incubated with 0 ng/mL ATTO488-NF were compared to the ATTO488 signal in cell populations incubated with 8.4, 17, 34 and 68 ng/ml of ATTO488-NF. For both the C2C12 and the RAW264.7 cell lines, all cell populations incubated with ATTO488-NF tested significantly different (<99% confidence) with respect to ATTO488 fluorescence from cell populations that were not incubated with ATTO488-NF.

Cellular uptake was demonstrated by the use of ATTO488-NF. 8.4 ng/ml of the labeled NF was incubated with cultured cell lines derived from macrophages or non-macrophage cell types, before potential uptake was analyzed by live-cell imaging using confocal microscopy. Representative images obtained when analyzing the murine cell lines C2C12 and RAW264.7 representing a myoblast and a macrophage cell line, respectively, are shown in Figure 2A. As evident from the microscopic pictures, cellular uptake of ATTO488-NF (visualized in green color) was observed upon incubation of ATTO488-NF with the macrophage-derived cell line (Figure 2A, lower right panel). Similar results were obtained when incubating the ATTO488-NF with human macrophages (THP-1 macrophages obtained by differentiation of THP-1 monocytes as described in (40)) as evident from Supplementary Figure S2. In both the murine and human macrophages, the uptake of ATTO488 strictly depended on its incorporation in the NF, and uptake of ATTO488-labeled nucleotides was not observed (Supplementary Figure S2). This is consistent with previous observations of cellular uptake only of assembled DNA octahedral structures and not labeled oligonucleotides (17) and most probably reflect repulsion of unstructured (poly)nucleotides from the negatively charged cell membrane (44) as mentioned in the introduction. Once inside the cells, the NF was excluded from the nucleus (visualized by Hoechst staining, blue color) and accumulated in well-defined compartments in the cytoplasm (Figure 2A and Supplementary Figure S2).

The macrophage specific uptake of ATTO488-NF was confirmed by flow cytometric analysis following incubation of macrophage- or non-macrophage-derived cell lines with the labeled NF (depicted as histograms in Figure 2B, Supplementary Figure S3 and dot plots in Supplementary Figure S2). As evident from Figure 2B the results of flow cytometric analysis were consistent with the results of the microscopic analyses and demonstrated that a markedly larger percentage of the macrophage derived RAW264.7 cells (4–97%) was ATTO488 positive compared to myoblast (C2C12) cells (1–25% positive) after incubation with increasing concentrations (ranging from 8.4 to 68 ng/ml) of ATTO488-NF. Likewise, we observed only modest uptake of ATTO488-NF in the non-macrophage, human embryonic kidney-derived (HEK293T) cells, fibroblast derived NIH/3T3 cells, and undifferentiated THP-1 monocytes (Supplementary Figure S3). These results are consistent with previous reports from the Ahn and Tan groups demonstrating none or only modest uptake of NFs in A549 lung adenocarcinoma cells, HepG2 liver cancer cells, K562 lymphoma and Ramos lymphoma cancer cells in the absence of functionalization that directly targeted the NFs to such cells (37,45). We did, however, observe a high level of uptake in the cervical cancer-derived (HeLa-CCL2) cells (Supplementary Figure S3). These cells resemble macrophages by the high expression level of scavenger receptors (46). Scavenger receptors were previously demonstrated essential for receptor mediated uptake of SNAs (15,16), an octahedral DNA structure (17), DNA origami structures (47) and plasmid DNA (48). Consequently, the uptake of NF in the non-macrophage HeLa-CCL2 cells may be explained by the high expression level of scavenger receptors in this cell line. As expected, and in concordance with previous reports on DNA structures, the NF was non-toxic to all cell lines investigated (Supplementary Figure S4) (8,37,49).

### Cellular uptake, localization and stability of the NF

**Figure 3.**
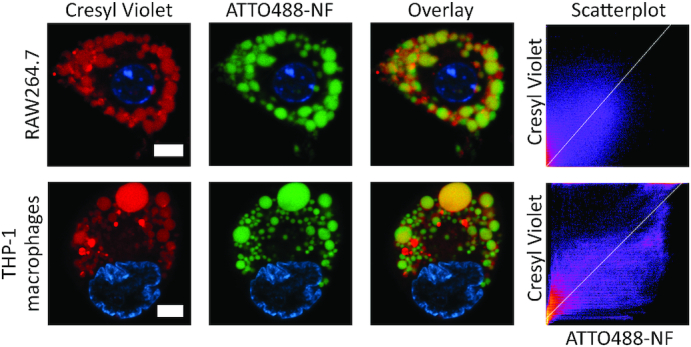
Lysosomal localization study using ATTO488-NF and Cresyl Violet stained lysosomes in RAW264.7 (upper panel) and THP-1 macrophages (lower panel). The pictures in the column denoted Cresyl Violet show Hoechst-stained nucleus (blue) and lysosomes (red). Pictures in the column denoted ATTO488-NF show the intracellular location of ATTO488-NF (green) and the nucleus (blue). In the column denoted Overlay, green fluorescence from ATTO488-NF is superimposed with the red fluorescence from Cresyl Violet and co-localization appears yellow. The white scale bars are 10 μm. The scatterplots to the right show a quantification of the co-localization between ATTO488-NF and Cresyl Violet in the depicted cells. The white line in the scatterplots is a linear regression of the co-localization, where perfect co-localization will yield a diagonal line from the bottom left to the top right in the scatterplot. Pearson's *R* values for the co-localization in the RAW267.4 and THP-1 macrophages are 0.41 and 0.78, respectively.

As mentioned above, ATTO488-NF accumulated in well-defined compartments in the cytoplasm of macrophages. To investigate the cellular localization of the NF more precisely, macrophages were incubated with ATTO488-NF, before the lysosomes and nuclei of RAW264.7 and THP-1 macrophages were stained using Cresyl Violet and Hoechst resulting in red and blue fluorescence, respectively (Figure 3 and Supplementary Figure S5). The cells were analyzed by live cell imaging using a confocal microscope. Cresyl Violet stained well-defined vacuoles in the cytoplasm in both cell lines (column denoted Cresyl Violet), previously demonstrated to be lysosomes (50). ATTO488-NF accumulated in well-defined vacuoles (column denoted ATTO488-NF) that overlapped with the Cresyl Violet stained lysosomes as evident from the overlay of the green and red channels (column denoted Overlay). ATTO488-NF localizing in the lysosomes was furthermore quantified using Coloc2 (ImageJ). This analysis is shown in the scatterplot to the right in Figure 3. These results strongly suggest that ATTO488-NF accumulated in the lysosomes of the macrophages. Such lysosomal localization is in agreement with previous studies demonstrating accumulation of pristine DNA structures such as an origami structure, a truncated octahedron and a tetrahedral DNA structure in lysosomes of COS, HEK, HeLa, MCF-7 and MCF-10A cells (17,23,26).

The uptake and intracellular localization of the NF in individual macrophages were followed by time-lapse microscopy demonstrating the appearance of intracellular fluorescent dots, most probably representing lysosomal accumulation, at 1 h after addition of ATTO488-NF (Supplementary Figure S6A). This is consistent with the results of confocal microscopy analysis of macrophages at different time intervals ranging from 1 to 16 h after uptake (data not shown) that showed a clear compartmentalization of the NF after one hour.

We used flow cytometric analysis to obtain information on the average time of maximum uptake of the ATTO488-NF in a RAW264.7 cell population. As evident from Supplementary Figure 6B, we observed an increase of ATTO488-NF uptake until a plateau was reached after an incubation time of eight hours even though the ATTO488-NF was stable in serum for up to 48 h (see Supplementary Figure S7A). Hence, the incubation period to obtain maximum uptake likely reflects saturation of the cells rather than degradation of ATTO488-NF in the media. Consequently, subsequent uptake experiments were performed using an 8-h incubation period. The intracellular stability of the ATTO488-NF was addressed in terms of lysosomal degradation monitored in terms of loss of fluorescence following the same principle as in the original lysosomal activity assays described by Mego *et al.* (51). After initial uptake of ATTO488-NF in RAW264.7 cells, excess ATTO488-NF was removed from the media and the amount of intracellular fluorescence measured by flow cytometry and confocal microscopy as a function of time. As evident from Supplementary Figure S7B, the half-life of ATTO488-NF was estimated to be approximately 12 h inside macrophages.

This result is consistent with an intracellular decay between 2 and 24 h of an octahedral DNA cage (17) but less than a tetrahedral DNA structure, which have been shown to remain stable in cells for up to 48 h (20). The relatively fast degradation of the NF compared to the rigid tetrahedral structure may be caused by a more flexible structure making the NF more susceptible to enzymatic and chemical degradation.

### Co-localization of ATTO488-NF and pathogens in infected macrophages

Given the intracellular localization of the NF in lysosomes it is possible that it will co-localize with pathogens that escape the human immune system by hiding in the lysosomes and, hence, be a putative drug carrier for treatment of such pathogen caused diseases.

We investigated whether the ATTO488-NF co-localized with pathogens inside infected macrophages by using two very different model pathogens; the leishmaniasis causing protozoan *L. infantum* and the tuberculosis causing bacteria, *M. tuberculosis*. The reason for this choice was that *L. infantum* and *M. tuberculosis* utilize different mechanisms to escape lysosomal degradation. *L. infantum* belongs to the herein denoted group 2 intracellular pathogens, which delay phagosomal maturation, whereas *M. tuberculosis* belongs to the group 1 pathogens, which prevent fusion between phagosomes and lysosomes (32).

**Figure 4.**
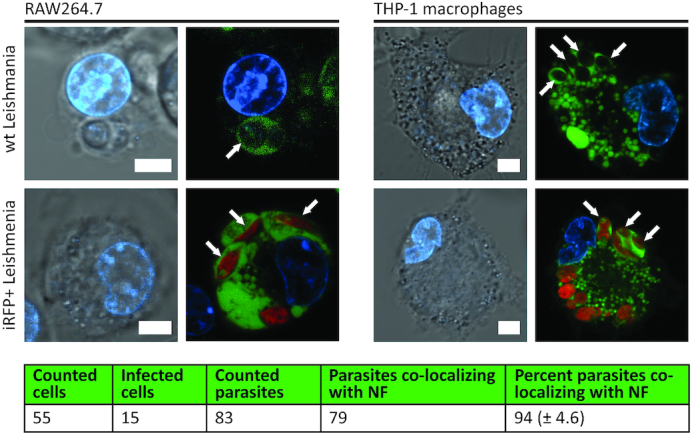
Co-localization analysis of ATTO488-NF in RAW264.7 (left) and THP-1 macrophages (right) infected with *L. infantum*. For each cell line, the left pictures are bright field pictures with the Hoechst stained nucleus superimposed (blue). In the upper pictures, the macrophages have been infected with wild-type *L. infantum* and in the lower pictures the macrophages have been infected with iRFP+ *L. infantum*. For each cell line, the picture to the right shows fluorescence from the nucleus and ATTO488-NF (green). In the pictures, wild-type *L. infantum* is colorless, whereas iRFP+ *L. infantum* is indicated in red. The white scale bars in the bright field microscopy pictures are 10 μm and the white arrows point at compartments with a clear co-localization between ATTO488-NF and *L. infantum*. The table shown in the lower panel summarizes a quantification of the co-localization observed in confocal microscopy between iRFP+ *L. infantum* and ATTO488-NF. The table shows the total number of cells counted, the number of infected cells, the number of parasites and the number of parasites that co-localize with the NF. The average percent of parasites co-localizing with ATTO488-NF from three experiments is calculated and the standard deviation is indicated in brackets.

Co-localization of the ATTO488-NF and *L. infantum* in infected macrophages was investigated by first infecting RAW264.7 and THP-1 macrophages. Six hours after infection, the cells were incubated 8 h with the labeled NF before analysis in a confocal microscope. The incubation time of infected cells with the labeled NF was chosen to ensure maximum cellular uptake of the NF (Supplementary Figure S6B). As model pathogens we used wild-type (strain BCN 150) (40) or a gene modified (iRFP+) *L. infantum* strain. The latter stably expresses the infrared fluorescent protein (iRFP) (40). The results of confocal-microscopy analysis are shown in Figure 4 and Supplementary Figure S8. As evident from the Figure 4, the green fluorescent ATTO488-NF co-localize with both wild-type *L. infantum* (evident as black shadows in the top row (marked with white arrow)) and infrared fluorescent parasites (shown in red in the lower row (marked with white arrow)) in the phagolysosomes of both infected RAW264.7 (left panels) and THP-1 macrophages (right panel). The co-localization between the labeled NF and the infrared *L. infantum* may be even more clear in the movie shown in Supplementary Figure S9.

**Figure 5.**
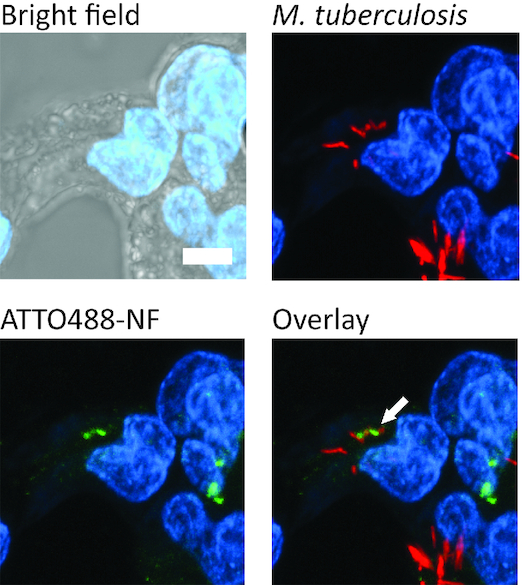
Co-localization analysis of ATTO488-NF in THP-1 macrophages infected with *M. tuberculosis* expressing Td tomato fluorescent protein. In the upper left corner is the bright field picture with Hoechst stained nucleus (blue) superimposed. The lower left corner is a confocal picture with nucleus and ATTO488-NF (green). The upper right picture shows the nucleus and *M. tuberculosis* (red). The lower right picture shows ATTO488-NF superimposed on the *M. tuberculosis*. The white scale bar is 5 μm and the white arrow points at co-localization between ATTO488-NF and *M. tuberculosis*.

The potential co-localization of the ATTO488-NF with *M. tuberculosis* was investigated in THP-1 macrophages 6 h post infection with the *M. tuberculosis* strain H37Rv that could be visualized due to overexpression of Td tomato fluorescent protein. The infected cells were incubated with ATTO488-NF as described above and analyzed by confocal microscopy. As evident from the resulting microscopic images (Figure 5 and Supplementary Figure S10), *M. tuberculosis* (visualized by red fluorescence) and ATTO488-NF (green fluorescence) co-localized in the infected cells (overlay).

## DISCUSSION

In the current study, we demonstrate the preferred uptake of an ATTO488 labeled NF in mammalian macrophage derived cell lines, while uptake was not observed in cell lines derived from an array of other cell types, except of HeLa-CCL2. HeLa-CCL2 cells, however, are characterized by an unusual (for a non-macrophage) high expression of scavenger receptors, which most probably explain the uptake of NF in these cells. Note that the cellular uptake of a pristine octahedral nanocage as well as SNA structures was previously demonstrated to depend on the expression of scavenger receptors (15–17). It was argued that these structures resemble the natural substrates of several scavenger receptors in both size (∼20 nm), shape, and charge density and that such resemblance could account for their efficient scavenger receptor mediated internalization. Although the NF is considerably larger (200–300 nm) than SNAs and the reported octahedral nanocage, it resembles these structures with respect to shape and charge density and is most probably internalized via scavenger receptors that are highly expressed in macrophages (46). Note, that internalization of larger particles via scavenger receptors has been reported (52,53).

Inside the macrophages, the NF was compartmentalized within a 1-hour time frame and was shown to concentrate in phagolysosomes. Yet, they remained relatively stable with an intracellular half-life of 12 h. The intracellular localization of the NF inspired us to investigate if the DNA structure co-localized with some of the widespread human pathogens *L. infantum* and *M. tuberculosis* that reside inside the macrophage phagolysosomes and, therefore, are difficult to treat with conventional drug formulations. These pathogens escape the human immune response by preventing important macrophage-mediated microbicidal functions including several signaling pathways and the phagocyte oxidative burst. Moreover, both pathogens affect phagosome maturations although in two different ways (54). *M. tuberculosis* block maturation by targeting key regulatory pathways. In addition, it secretes protein tyrosine phosphatase A, which interferes with late endosome/lysosome vesicular interactions. Leishmania do not arrest but rather delay phagosome maturation using a surface-associated lipophosphoglycan. Despite such modulations of phagolysosome biology, we observed a clear co-localization between the ATTO488-NF and both *L. infantum* and *M. tuberculosis* in infected macrophage cells. This suggests a potential application of the NF as a drug carrier for targeted treatment of diseases caused by these and alike pathogens. Further supporting such applications, several groups demonstrated the possibilities of coupling relevant small molecule drugs (e.g. doxorubicin) to DNA structures (8,37,55) and pH dependent drug release from NFs (37,45). Doxorubicin has a well-documented anti-leishmania effect (36,56). However, treatment is often inefficient due to the relatively slow cell doubling of Leishmania parasites inside macrophages. Delivery of a high local concentration of doxorubicin carried by the NF combined with a prolonged release facilitated by conformational changes in the acidic environment or slow degradation is likely to improve treatment outcome considerable. Tuberculosis is treated with a combination of small molecule drugs including isoniazid, rifampin, ethambutol, pyrazinamide (Treatment for TB Disease. https://www.cdc.gov/tb/topic/treatment/tbdisease.htm, 57), which may also be coupled to the NF for prolonged drug release.

During the past decades the development of new and effective drug delivery systems for treatment of infectious diseases caused by macrophage residing pathogens have received much attention. Accordingly, various delivery systems have been investigated with various degree of success although only few of them have reached the market. This may be due at least in part to an inherent toxicity associated with such structures combined with relative high production costs (35,36). NF structures are characterized by a high degree of biocompatibility, they are non-toxic and may even be produced at high scale at low cost by e.g. bacteriophages (58,59). Hence, they may represent an attractive alternative for future targeting of intracellular pathogens. Moreover, compared to more traditional drug carriers such as liposomes, NFs are relatively easy to modify with regard to factors such as size, shape and functionality that may affect the cellular uptake (10,11,37,45). As demonstrated in the current study NFs are also characterized by a relatively high intracellular stability, which may be crucial for effective treatment of tenacious pathogens such as *M. tuberculosis* and *L. infantum*.

We believe that the co-localization of ATTO488-NF with the two pathogens, which exploit very different mechanisms to survive the hostile environment inside macrophages, hold promise for the use of NFs as versatile drug carriers for targeting diseases caused by pathogens that are able to fool the human immune system by hiding in the macrophage. Such pathogens are the cause of some of the most serious infectious diseases worldwide, not least due to the difficulties of effective treatment. For instance, the duration of tuberculosis treatment is typically between 6 and 12 months (60) and even longer for the multi-drug-resistant forms, while visceral leishmaniasis typically requires hospitalization for up to a month (36). Consistently, multidrug-resistant microbes (including *M. tuberculosis* and Leishmania spp.) are on the WHO list of global heath treat (33 (https://www.who.int/emergencies/ten-threats-to-global-health-in-2019)).

## Supplementary Material

gkaa341_Supplemental_Files
