# Supplementary material for: DNA flowerstructure co-localizes with human pathogens in infected macrophages
Source: Nucleic Acids Res. 2020 May 13;48(11):6081–91. doi: 10.1093/nar/gkaa341 (PMC7293011; doi:10.1093/nar/gkaa341)
Supplement: gkaa341_Supplemental_Files [file gkaa341_supplemental_files.zip › 21042020_Franch et al_Supplementary data.pdf]

Supplementary data

DNA Flowerstructure co-localizes with Human Pathogens in Infected Macrophages

Oskar Franch[1,3], Camino Gutiérrez-Corbo[2], Bárbara Domínguez-Asenjo[2], Thomas Boesen[3,4], Pia Bomholt Jensen[3], Lene N. Nejsum[5], Josephine Geertsen Keller[1,5], Simon Pagaard Nielsen[1], Prakruti R. Singh[6], Rajiv Kumar Jha[6], Valakunja Nagaraja[6], Rafael Balaña-Fouce[2], Yi-Ping Ho[7,8], Rosa María Reguera[2], Birgitta Ruth Knudsen[1,3#]

[1] Department of Molecular Biology and Genetics, Aarhus University, Aarhus, Denmark

[2] Department of Biomedical Sciences, University of León, León, Spain.

[3] Interdisciplinary Nanoscience Center (iNANO), Aarhus University, Aarhus, Denmark

[4] DANDRITE, Nordic EMBL Partnership for Molecular Medicine, Department of Molecular Biology and Genetics, Aarhus University, Aarhus, Denmark.

[5] Department of Clinical Medicine, Aarhus University, Aarhus, Denmark

[6] Department of Microbiology and Cell Biology, Indian Institute of Science & Jawaharlal Nehru Centre for Advanced Scientific Research, Bangalore, India

[7] Department of Biomedical Engineering, The Chinese University of Hong Kong, Hong Kong SAR

[8] Centre for Novel Biomaterials, The Chinese University of Hong Kong, Hong Kong SAR

[#]To whom correspondence should be addressed. E-mail: brk@mbg.au.dk, phone: +45-60202673

Keywords: DNA nanostructure, Macrophage residing pathogens, DNA Nanoflower, *Mycobacteria tuberculosis*, *Leishmania infantum*

Dynamic Light Scattering confirms the NF size of approximately 300 nm

Dynamic Light Scattering (DLS) analyses was performed to address the size of NF in solution. The DLS measurements were performed on an ALV instrument (ALV, Langen, Germany) with an ALV/CGS-8F goniometer equipped with an ALV-6010/EPP multi-tau digital correlator. The instrument operates with a wavelength of 632.8 nm in the pseudo-cross correlation mode and the measurements were performed at a 90° scattering angle. The data were fitted with a double schultz distribution of decay rates (1). The polydispersities were quite large, however, without deteriorating the fits significantly, the relative polydispersities were kept, respectively, at 0.7 for the small size contribution and 0.4 for the larger size contribution. This corresponds to polydispersity indices (PDI) of respectively, 0.49 and 0.16.

Fig. S1 shows the DLS analysis of the NF. The DLS analysis reveals two populations in the analysis, one with a hydrodynamic size of approximately 15 nm and the other with an approximately size of 324 nm. The particles of 15 nm correspond to the sizes Bovine Serum Albumin (BSA) (2) and phi29 (3), which are proteins used to create NF. The particles of around 300 nm also matches the size of the NF observed under imaging with a TEM (See Fig. 1 in the main text).

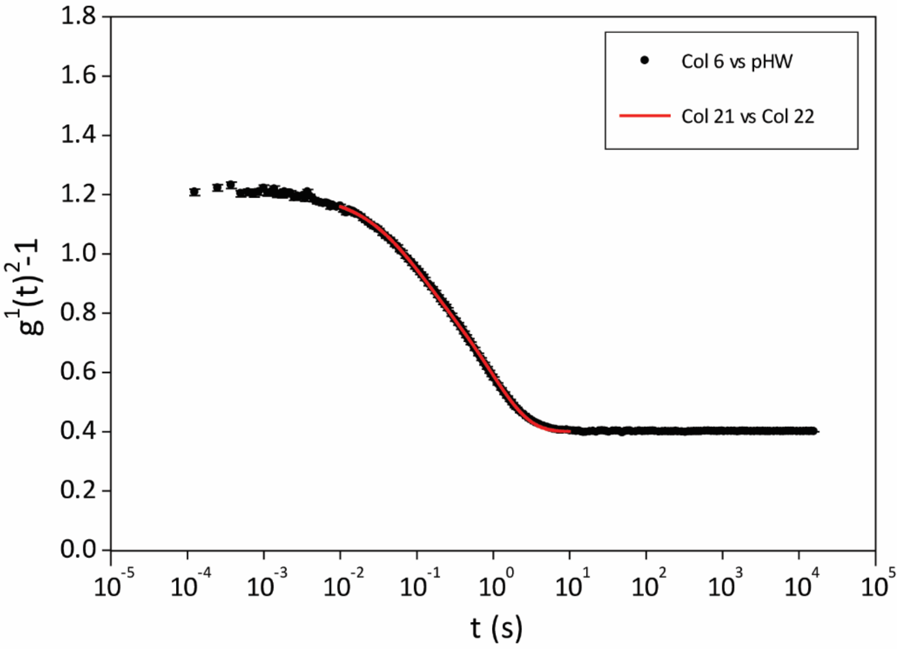

Double Schultz			D_H	σ		Factor	Mass	%(mass)	$\chi 2$
$\Gamma 1$	10.16	=>	15 nm	0.7	Scale1	0.2241	0.000558	99.97	1.02

$\Gamma 2$	0.4634	=>	324 nm	0.4	Scale2	0.6718	1.59E-07	0.03	

Figure S1 Graph: The DLS data in black dots fitted with a Double Schultz distribution of decay rate (red line). The table below show parameters revealed from the DLS analysis, including the hydrodynamic size (D_H) of the particles.

ATTO488-NF is taken up by macrophages

The uptake of ATTO488-NF in murine RAW264.7 macrophages and human THP-1 macrophages was addressed using confocal microscopy analyses. In addition, background fluorescence from unincorporated ATTO488-dUTP that remain after purification of the ATTO488-NF was addressed. Fig. S2 (top panel) shows confocal microscope pictures of RAW264.7 macrophages (left) and THP-1 macrophages (right) that have been incubated with 4.2 ng/mL ATTO488-NF (bottom pictures). In addition, the background of free ATTO488-dUTP was addressed (top pictures) by incubating the cells with a negative sample prepared as the ATTO-488-NF sample except that addition of phi-29 polymerase was omitted.

The results illustrate that the background from unincorporated ATTO488-dUTP is dismissible in comparison to the signal from ATTO488-NF. Fig. S2 (top panel) also illustrate that ATTO488-NF is taken up in both the murine RAW264.7 macrophages and the humane THP-1 macrophages. We did not observe any apparent difference in the uptake between the two type of macrophages. Fig. S2 (bottom panel) show dot plot depiction (side scatter versus ATTO488 fluorescence) of the results shown in Fig. 2B in the main text.

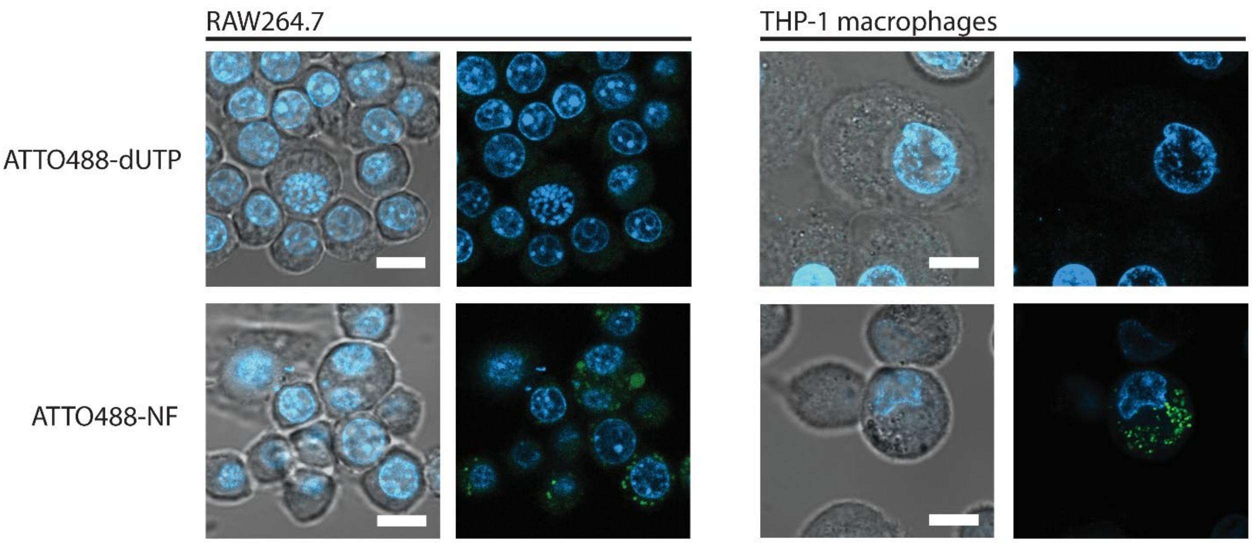

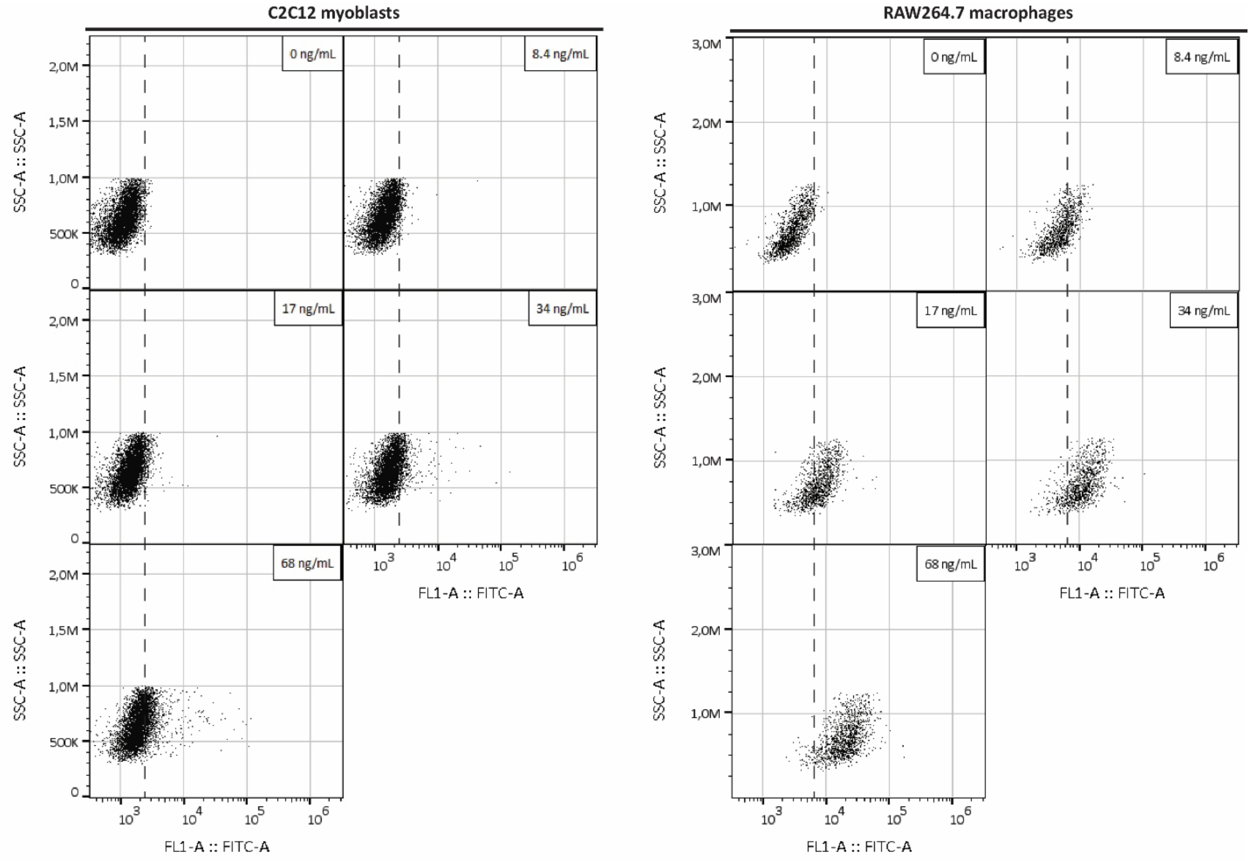

Figure S2 Top panel: The uptake of ATTO488-NF and ATTO488-dUTP in RAW264.7 (four left pictures) and THP-1 macrophages (four right pictures). For each cell line, the left pictures are bright field pictures with the Hoechst stained nucleus (blue) superimposed on the picture. The right pictures in each cell line, show fluorescence from Hoechst stained nucleus and ATTO488 (green) from either ATTO488-dUTP or ATTO488-NF. The upper panel depicts cells incubated with a negative sample prepared as the ATTO488-NF sample except that phi-29 polymerase was omitted to prevent formation of RCA product. The cells in the lower panel have been incubated with a sample containing ATTO488-NF. The white scale bars in the bright field microscopy pictures are 10 µm. Bottom panel: Show dot plots of side scatter (SSC-A) versus the ATTO488 fluorescence intensity (FITC-A) of the cell populations from Figure 2B in the main text. The five plots to the left show results from C2C12 myoblasts and the five plots to the right show RAW264.7 macrophages incubated with 0 ng/mL, 8.4 ng/mL, 17 ng/mL, 34 ng/mL and 64 ng/mL ATTO488-NF as indicated in the top right corners of the plots. The broken line illustrates the threshold for cells positive uptake as defined in the main manuscript.

In non-macrophagial cell lines, uptake of ATTO488-NF was only observed in HeLa-CCL2

Uptake of ATTO488-NF was studied in murine NIH/3T3 fibroblasts, human HEK293T cells, human HeLa-CCL2 epithelial and undifferentiated THP-1 monocytes using flow cytometry.

HEK293T (kindly provided by Associate Professor Pia Møller Martensen, Department of Molecular Biology and Genetics, Aarhus University) and HeLa-CCL2 were cultured in DMEM with 10 % FBS, 100 units/mL penicillin and 100 mg/mL streptomycin. NIH/3T3 (kindly provided by Professor Finn Skou Pedersen, Department of Molecular Biology and Genetics, Aarhus University) were cultured in DMEM supplemented with 10 % newborn calf serum, 100 units/mL penicillin and 100 mg/mL streptomycin. All cells lines were incubated in a humidified incubator (5 % CO_2) at 37 °C. The cells were seeded in a standard 6 well (Sarstedt) microtiter plate. After 24 hours, the cells were 80 % confluent and samples were added to the cells for a final concentration of 0 ng/mL, 4.2 ng/mL, 17 ng/mL, 34 ng/mL and 68 ng/mL of ATTO488-NF. After 16 hours of incubation, the samples were removed, and the cells were washed twice with PBS, before fluorescence in the cells were quantified using flow cytometry.

The vertical line through the histograms in Fig. S3 separate the cells positive for ATTO488 signal from cells negative for ATTO488 signal, as described in the main text. The flow cytometry analyses illustrate that 10 %, 9 % and 7 % of NIH/3T3 fibroblasts, HEK293T, and undifferentiated THP-1 monocytes, respectively, were fluorescently labeled after incubation with 68 ng/mL of ATTO488-NF. In contrast, 91 % of the HeLa-CCL2 cells were positive for ATTO488 signal after incubation with 68 ng/mL of ATTO488-NF. HeLa-CCL2 cells have epithelial morphology, but have upregulated levels of scavenger receptors like CLA-1, which is a scavenger receptor that is also upregulated in THP-1 macrophages (4).

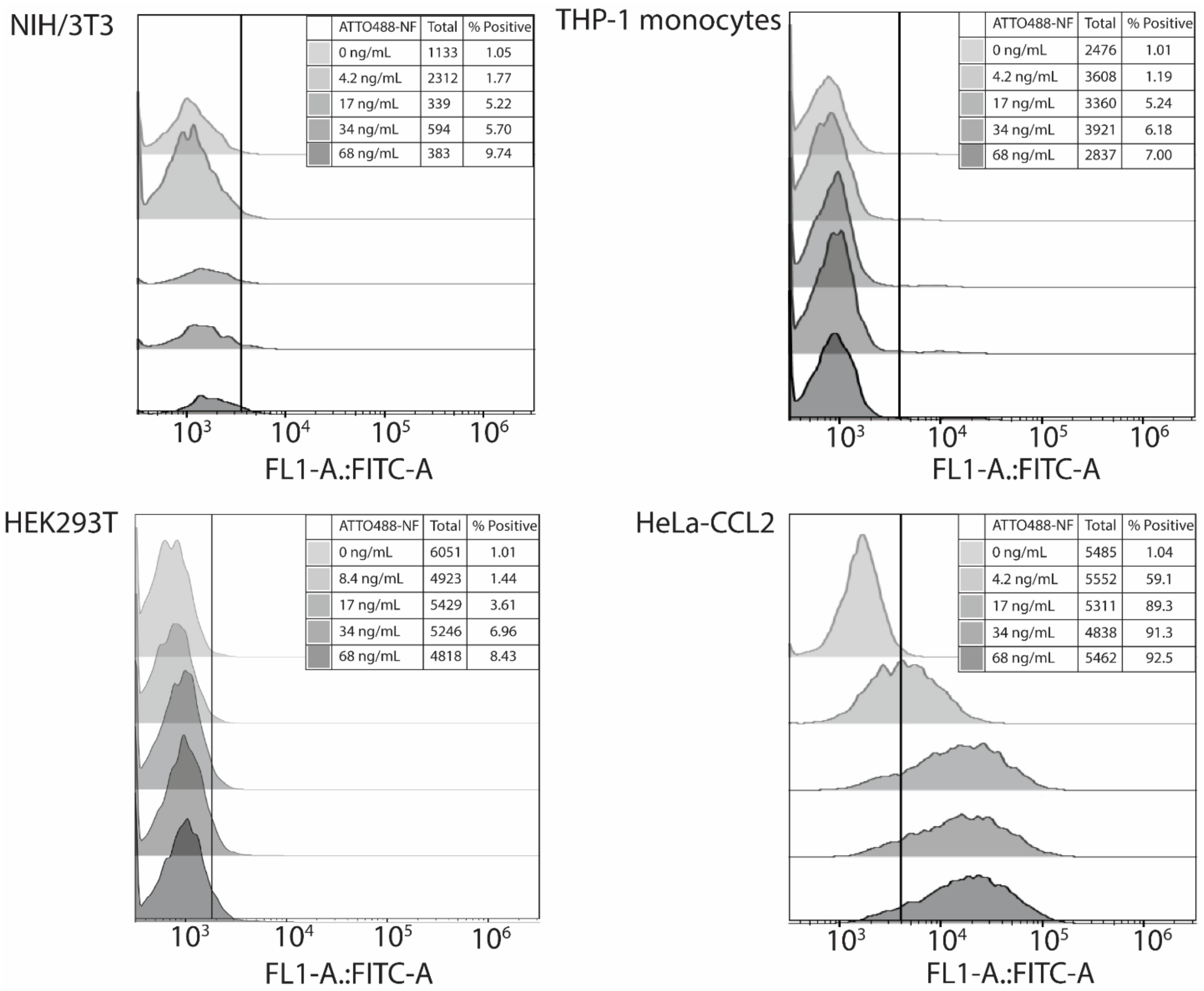

Figure S3. Flow cytometry analysis of uptake of ATTO488-NF in NIH/3T3, THP-1 monocytes, HEK293T and HeLa-CCL2. The first axis has a logarithmic scale and indicates the intensity of the ATTO488 signal, whereas the second axis indicates the number of cells. The vertical line through the histograms separate cells positive for ATTO488-NF uptake from cells negative for uptake. The tables in the top right corner of the histograms show the total number of cells represented in the histograms and the percent of cells positive for uptake.

The NF is non-toxic

Toxicity of the NF was addressed using RAW264.7 macrophages, C2C12 myoblasts, HEK293T fibroblast and NIH/3T3 fibroblasts.

Cells were seeded in 96-well flat bottom plates (Corning, Inc., Corning, NY) and incubated for 24 hours. After 24 hours, medium was replaced with medium containing 2000 ng/mL of NF or PBS (as a negative control). The cells were further incubated for 72 hours. Subsequently, 10 µL of PrestoBlue cell viability reagent (Invitrogen) was added and the cells were incubated for additionally 4 hours. The fluorescence emission was measured at 540 nm excitation/590 nm emissions using a FLUOstar OPTIMA (BMG Labtech, Ortenberg, Germany) microplate reader. After background subtraction, fluorescence values were normalized for percent survival relative to the cells incubated with PBS. Data were plotted as mean (6 wells) ± SD values.

The results are depicted in Fig. S4 and demonstrate that the NF did not exhibit toxicity in the examined cell lines.

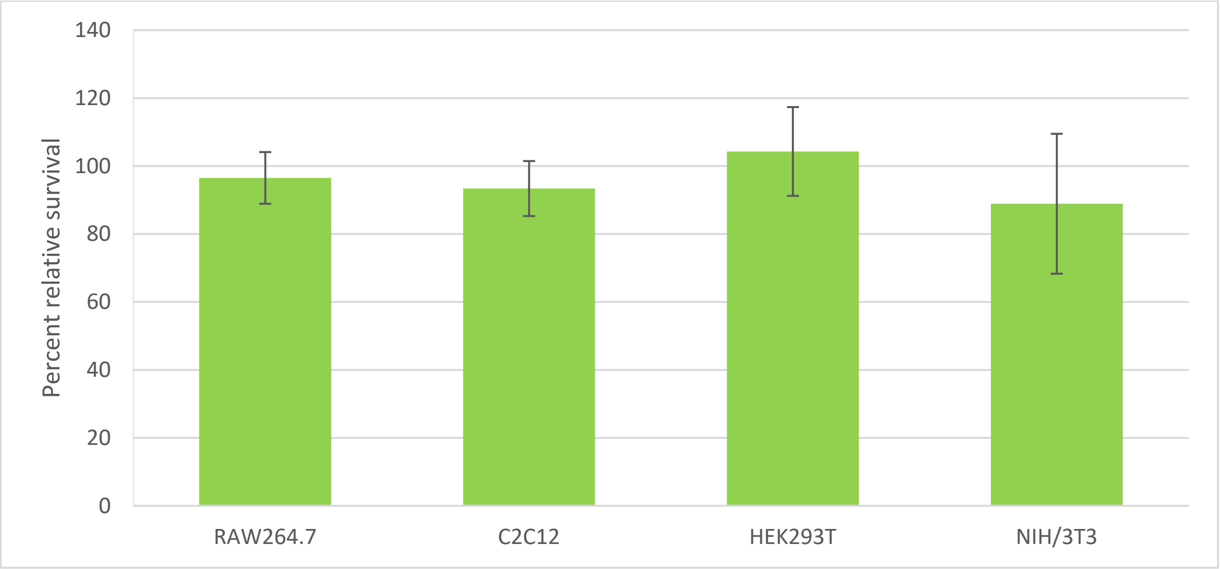

Figure S4. Illustrates the survival rate (percent relative to the PBS control) of RAW264.7 macrophages, C2C12 myoblasts, HEK293T fibroblast and NIH/3T3 fibroblasts incubated with 2000 ng/mL NF

ATTO488-NF accumulated in the lysosomes of the macrophages

The cellular localization of the NF in murine RAW264.7 macrophages and human THP-1 macrophages was addressed using confocal microscopy analyses.

Fig. S5 shows confocal microscope pictures of THP-1 macrophages (top two rows) and RAW264.7 macrophages (bottom two rows) that were incubated with 8.4 ng/mL ATTO488-NF, before the lysosomes and nuclei of RAW and THP-1 macrophages were stained using Cresyl Violet and Hoechst resulting in red and blue fluorescence, respectively. The cells were analyzed by live cell imaging using a confocal microscope as described in the main text. ATTO488-NF accumulated in well-defined vacuoles (middle column) that overlapped with the Cresyl Violet stained lysosomes as evident from the overlay of the green and red channels (right column).

These results strongly suggest that ATTO488-NF accumulated in the lysosomes of the macrophages.

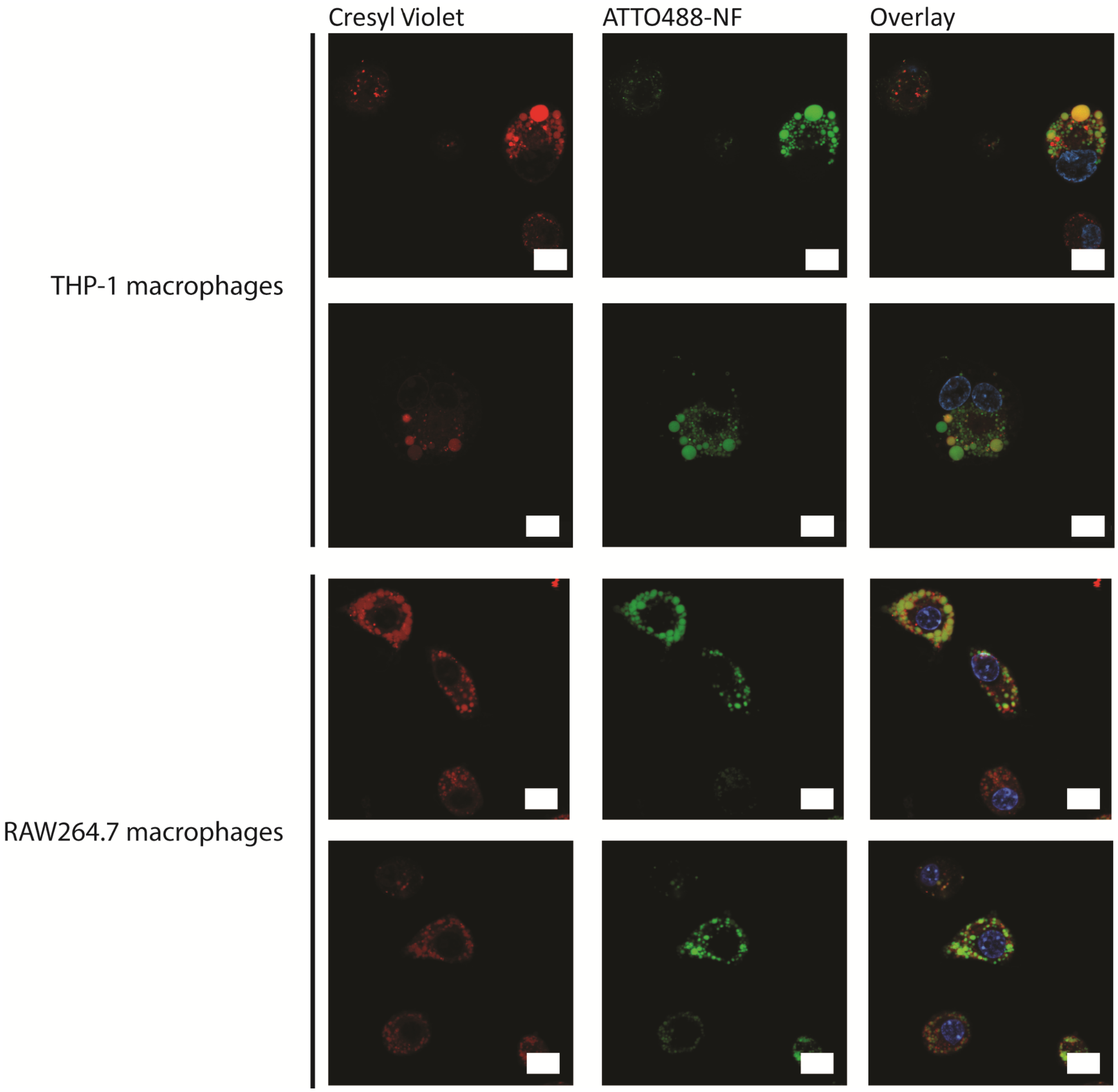

Figure S5. Lysosomal localization study using ATTO488-NF and Cresyl Violet stained lysosomes in THP-1 macrophages (top two rows) and RAW264.7 macrophages (bottom two rows). The pictures to the left show the lysosomes (red). Pictures in the middle column shown the intracellular location of ATTO488-NF (green). To the right, green fluorescence from ATTO488-NF is superimposed with the red fluorescence from Cresyl Violet and co-localization appears yellow. The white scale bars are 10 µm.

ATTO488-NF is internalized in RAW264.7 within 1 hour of incubation and internalization stalls after 8 hours

The uptake rate of ATTO488-NF in RAW264.7 was studied using time-lapse microscopy and flow cytometry.

RAW264.7 macrophages in an 8-well ibidi coated plate for the time-lapse microscopy or in standard 6-well plates for flow cytometry. After 24 hours, when the macrophages were 80 % confluent, ATTO488-NF was added to the cells for a final concentration of 8.4 ng/mL in both the time-lapse microscopy experiments and flow cytometry experiments. The time-lapse image sequences illustrate the initiation of uptake in macrophages and were obtained using a Nikon Ti Eclipse inverted microscope equipped with an OkoLab heating chamber, Perfect Focus 3 system, a 60x objective, and an Andor Zyla cMOS camera. Imaging was performed every 15 minutes for 12 hours using filters for Hoechst and FITC (ATTO488). The montage in Fig. S6A shows the sequence of time-lapse images of RAW264.7 macrophages incubated with ATTO488-NF from time 0 to 3.5 hours after addition of ATTO488-NF. After 15 minutes the top macrophage encounters a large NF that appears as a large, brightly green dot. Within 1 hour, the macrophage contained large, intracellular structures with ATTO488-NF. Likewise, the middle, right macrophage encounters an ATTO488-NF (visible in the 1-hour picture), which is internalized during the next 30 minutes. The nuclei were stained using Hoechst live cells.

Flow cytometry analysis in Fig. S6B revealed the time required for a saturated uptake of ATTO488-NF in RAW264.7 macrophages. For this analysis, the macrophages were washed and harvested 1 hour, 4 hours, 8 hours and 16 hours after the ATTO488-NF had been added. Fluorescence was measured by flow cytometry (Dako Coulter) with an excitation wavelength of 480 nm and an emission wavelength of 550 nm. The negative control (NC) was used to establish the background fluorescent in cells, which had not been incubated with ATTO488-NF. Data were processed with FlowJo software. Fluorescently labeled macrophages is denoted as a percentage of the total amount of macrophages measured in the analysis. The flow cytometry analysis shows that the fraction of fluorescently labeled RAW264.7 macrophages did not increase further after 8 hours of incubation with ATTO488-NF.

From the combined experiments, we conclude that uptake of ATTO488-NF is observed in RAW264.7 macrophages within one hour after adding the ATTO488-NF and uptake is saturated after 8 hours of incubation.

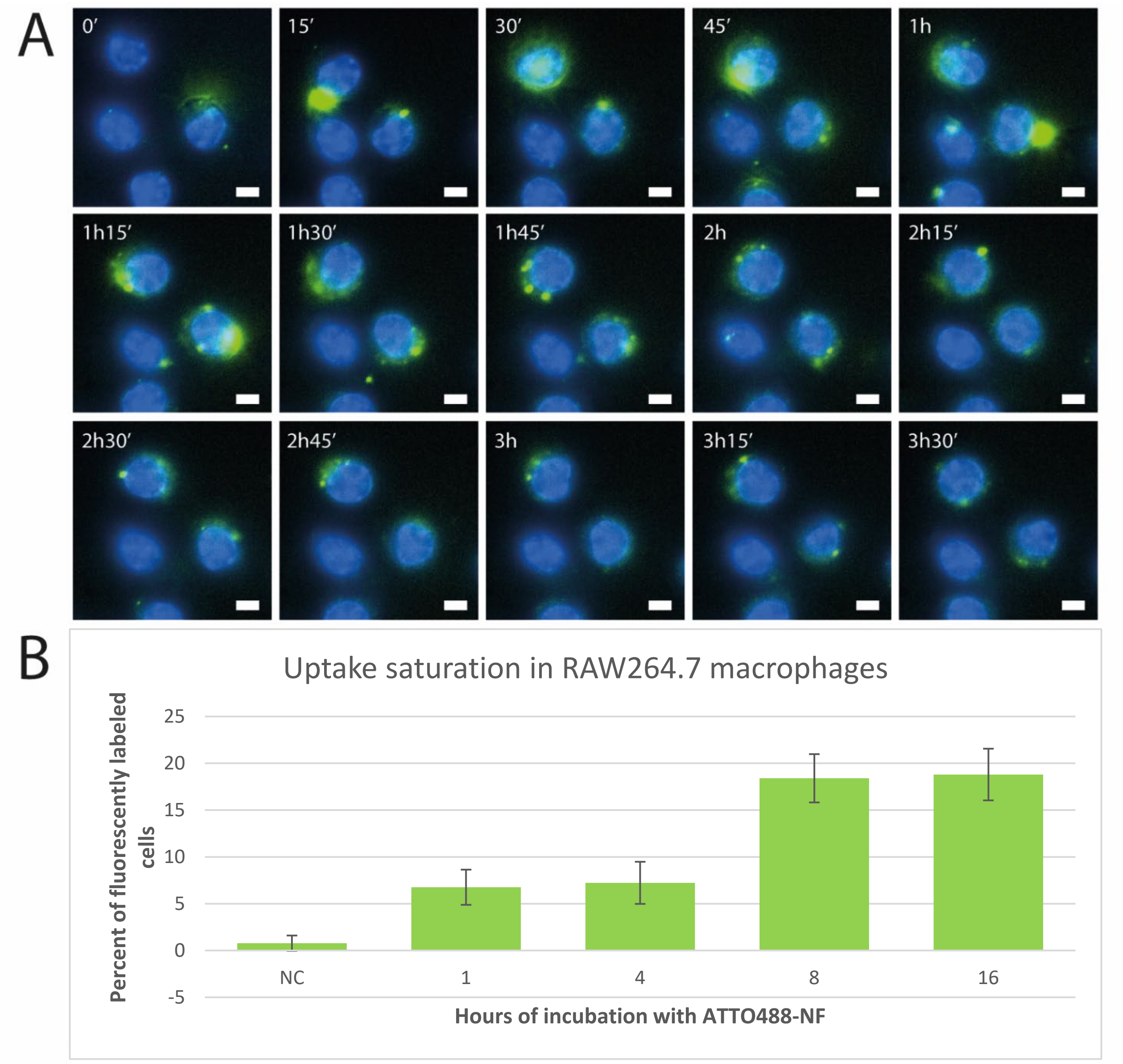

Figure S6. (A) Still images from a time-lapse image sequence obtained with 15 minutes intervals after adding ATTO488-NF to RAW264.7 macrophages. The nucleus is depicted in blue and the ATTO488-NF appears green. Scale bar in white indicate 5 µm. (B) Flow cytometry analysis of uptake of ATTO488-NF in RAW264.7 macrophages. Columns indicate RAW264.7 macrophages positive for uptake after incubating them either without ATTO488-NF (NC) or with ATTO488-NF

for 1 hour, 4 hours, 8 hours or 16 hours from left to right, respectively. Error bars represent standard deviation of the ATTO488 signal in the cell populations calculated using FlowJo software.

Stability of ATTO488-NF

The serum and intracellular stability of ATTO488-NF in RAW264.7 macrophages were addressed. The serum stability was measured by incubating 12.5 ng/mL ATTO488-NF with non-heat inactivated FBS for increasing time periods from 0 to 48 hours at 37 °C, reactions were stopped by adding 0.5% SDS (final concentration) and samples were stored at -20 °C. The amount of ATTO488-NF was estimated by hybridizing 5 µL of the samples diluted 100-fold to microscope slides functionalized with oligonucleotides having a sequence complementary to a 20-nucleotide region in the ATTO488-NF. Subsequently the number of signals representing individual NFs were counted using a fluorescent microscope. The results are depicted as bar charts in Figure S7A and demonstrate that the number of ATTO488-NF is unaffected by incubation with FBS for 48 hours. To investigate the intracellular stability of the NF, RAW264.7 macrophages were incubated with ATTO488-NF. The NF samples were removed from the cells 4 hours after incubation start, the cells were washed with PBS and new media added. Subsequently, the macrophages were incubated for 0 hours, 4 hours, 12 hours, 20 hours and 44 hours, before the fluorescence from ATTO488 in the macrophages were quantified using flow cytometry. In addition, a parallel experiment was performed using identical conditions to study the intracellular location of the ATTO488-NF using confocal microscopy.

The results, depicted in Figure S7B, demonstrate that fluorescence from ATTO488-NF in macrophages disappears after 44 hours and that ATTO488-NF has the half-life of 12 hours in macrophages. The disappearance of ATTO488 signal suggests that the macrophage degrade ATTO488-NF and excrete the metabolized entities. The pictures below the graph are from the confocal analysis that was performed in parallel. The pictures show that ATTO488-NF was present in vesicles inside the macrophages.

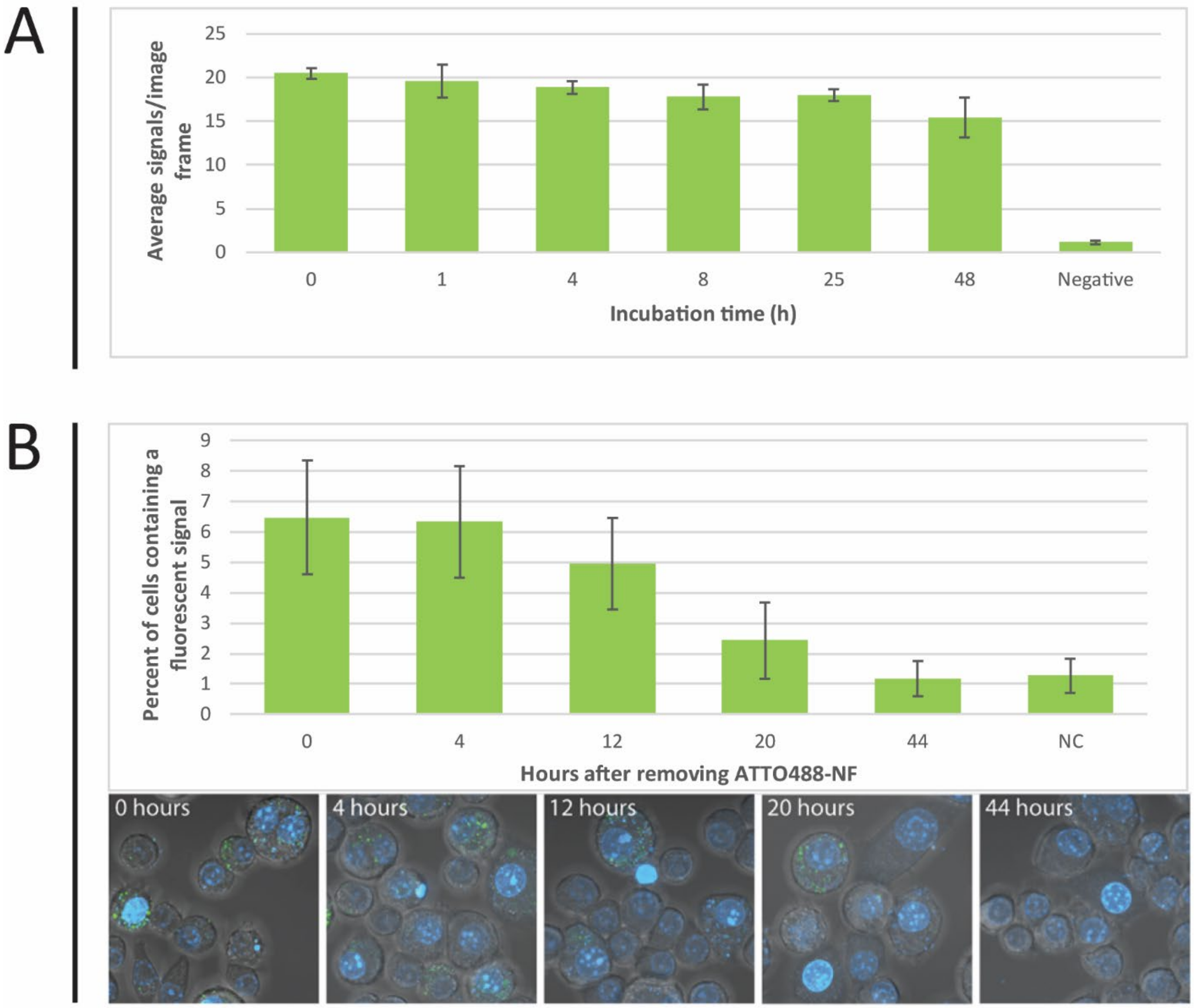

Figure S7. A. Bar chart showing the number of ATTO488-NF counted on a microscope slide after incubation with FBS for increasing time periods ranging from 0 to 48 hours. The bar marked negative shows the number of signals observed when no sample was added to the slide. B. The stability of ATTO488-NF in RAW264.7 macrophages. The bar chart depicts results from flow cytometry analysis. The height of the bars indicates the percentage of cells positive for uptake of ATTO488-NF. Each bar represents macrophages that have been incubated with 68 ng/mL of ATTO488-NF for four hours. Thereafter the sample was removed, the cells were washed and incubation was continued for the indicated hours. Error bars represent standard deviation of the ATTO488 signal in the cell populations calculated using FlowJo software. The pictures below show representative pictures of RAW264.7 cells with extended depth focus at the indicated hours after the NF-sample was removed.

ATTO488-NF co-localize with *L. infantum* in macrophages

Co-localization of the ATTO488-NF and *L. infantum* in infected macrophages was investigated. THP-1 macrophages and RAW264.7 macrophages were infected with the iRFP+ *L. infantum* strain. Six hours after infection, the cells were incubated with the labeled NF for eight hours before analyzed using a confocal microscopy. The iRFP+ *L. infantum* strain expresses the infrared fluorescent protein (iRFP). In Fig. S8, we show additional pictures similar to the examples presented in Fig. 4.

The pictures demonstrate that the green fluorescent ATTO488-NF co-localize with infrared fluorescent parasites (shown in red in the right column (examples marked with white arrow)) in the phagolysosomes of both infected THP-1 macrophages (top two rows) and RAW264.7 (bottom two rows).

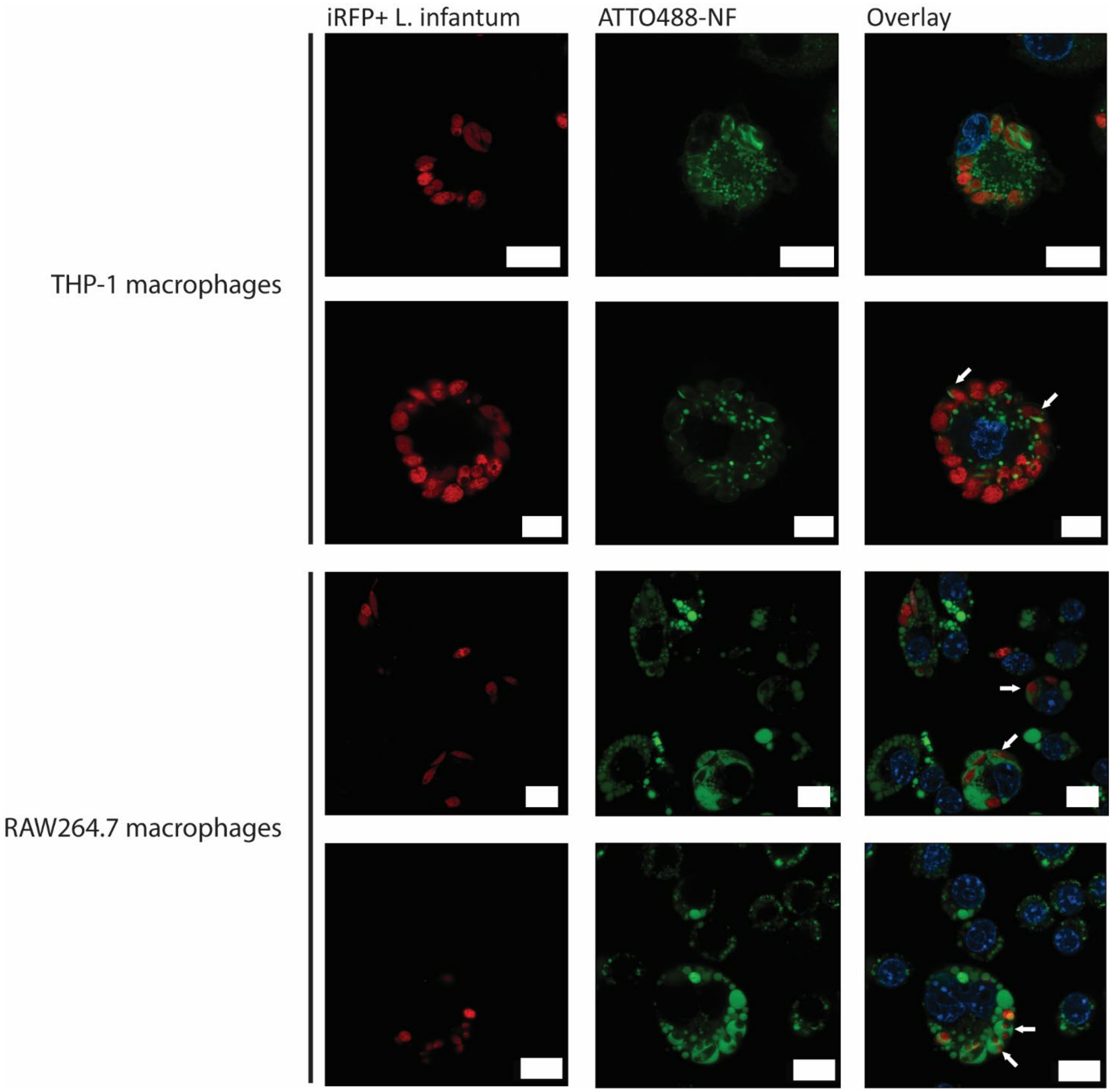

Figure S8 show representative pictures of the co-localization analysis between ATTO488-NF and infrared fluorescent parasites in the phagolysosomes of both infected THP-1 macrophages (top two rows) and RAW264.7 (bottom two rows). In the pictures shown in the left column fluorescence from iRFP+ *L. infantum* can be observed in red. Pictures in the middle column show fluorescence from ATTO488-NF (green). The right column depicts an overlay of the pictures shown in the left and middle column. The white scale bars are 10 µm and the white arrows point at examples of compartments with a clear co-localization between ATTO488-NF and *L. infantum*.

Footage of leishmania parasites whirling around in ATTO488-NF in vesicles inside an infected macrophage

RAW264.7 macrophages were cultured and infected as described in main text.

24 hours after the macrophages were infected, ATTO488-NF was added to the macrophages for a final concentration of 68 ng/mL. The macrophages were subjected to confocal analysis, after the macrophages had been incubated for 8 hours. The video was obtained with a handheld device and shows the computer screen during the confocal microscopy analysis.

Figure S9. A video of iRFP+ *L. infantum* co-localizing with ATTO488-NF in RAW264.7 macrophages. Initially, footage of light microscopy is observed, where the morphology of the RAW264.7 is observed. Next ATTO488-NF (green) is observed in large vesicles in the macrophage. Noticeable, dark shadows move inside two of the vesicles - one contains a single shadow, the other contain numerous. Lastly, the footage of the iRFP+ labeled parasite (red) is observed, where it becomes clear that the parasites co-localize with ATTO488-NF in the vesicles of the macrophage.

ATTO488-NF co-localize with *M. tuberculosis* in macrophages

The potential co-localization of the ATTO488-NF with *M. tuberculosis* was investigated in THP1 macrophages with the *M. tuberculosis* strain H37Rv.

THP-1 macrophages were infected with the *M. tuberculosis* strain H37Rv, as described in the main text. H37Rv was visualized via fluorescent protein. Fig. S10 show additional pictures to the results presented in Fig. 5.

The pictures demonstrate that the green fluorescent ATTO488-NF co-localize with fluorescent *M. tuberculosis* (shown in red) in the infected THP-1 macrophages. Examples of the co-localization is marked with white arrow.

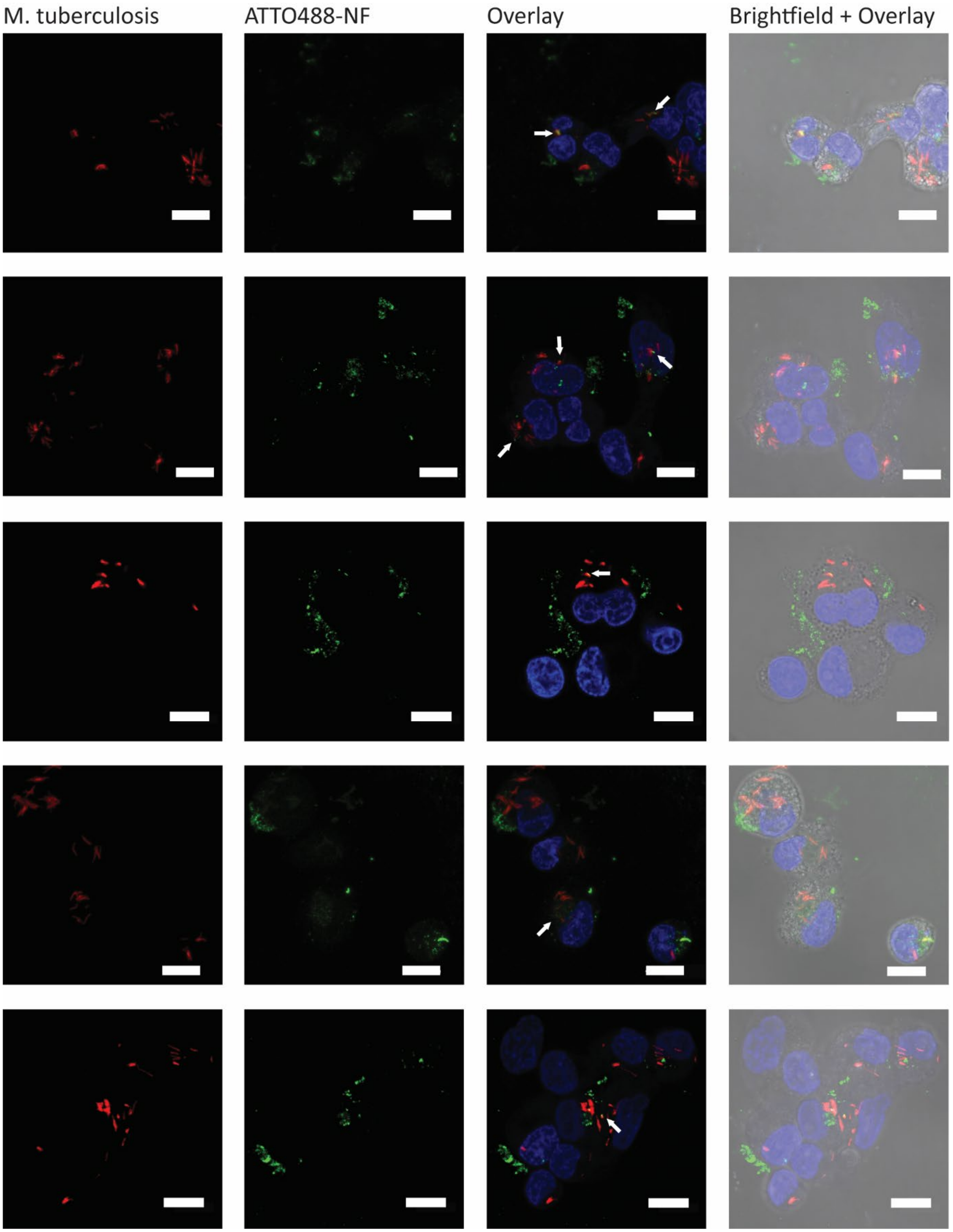

Figure S10. Co-localization analysis of ATTO488-NF in THP-1 macrophages infected with *M. tuberculosis* expressing Td tomato fluorescent protein. The columns denoted *M. tuberculosis* and

ATTO488-NF show confocal pictures with *M. tuberculosis* (red) and ATTO488-NF (green), respectively. The column denoted Overlay shows channel with *M. tuberculosis* (red) superimposed with the channel containing ATTO488-NF (green) and the nucleus (blue). The final column denoted Bright-field + Overlay, shows the bright-field picture overlaid with the fluorescence from channels containing *M. tuberculosis*, ATTO488-NF and the nucleus. The white scale bar is 10 µm and the white arrow points at co-localization between ATTO488-NF and *M. tuberculosis*.

References:

1. Mailer, A.G., Clegg, P.S. and Pusey, P.N. (2015) Particle sizing by dynamic light scattering: non-linear cumulant analysis. *J Phys Condens Matter*, **27**, 145102.
2. Wright, A.K. and Thompson, M.R. (1975) Hydrodynamic structure of bovine serum albumin determined by transient electric birefringence. *Biophys J*, **15**, 137-141.
3. Kamtekar, S., Berman, A.J., Wang, J., Lazaro, J.M., de Vega, M., Blanco, L., Salas, M. and Steitz, T.A. (2004) Insights into strand displacement and processivity from the crystal structure of the protein-primed DNA polymerase of bacteriophage phi29. *Mol Cell*, **16**, 609-618.
4. Baranova, I.N., Vishnyakova, T.G., Bocharov, A.V., Kurlander, R., Chen, Z., Kimelman, M.L., Remaley, A.T., Csako, G., Thomas, F., Eggerman, T.L. *et al.* (2005) Serum amyloid A binding to CLA-1 (CD36 and LIMPII analogous-1) mediates serum amyloid A protein-induced activation of ERK1/2 and p38 mitogen-activated protein kinases. *J Biol Chem*, **280**, 8031-8040.
